# Feasibility of a Digital Patient–Provider Communication Intervention to Support Shared Decision-Making in Chronic Health Care, InvolveMe: Pilot Study

**DOI:** 10.2196/34738

**Published:** 2022-04-07

**Authors:** Berit Seljelid, Cecilie Varsi, Lise Solberg Nes, Kristin Astrid Øystese, Elin Børøsund

**Affiliations:** 1 Department of Digital Health Research Division of Medicine Oslo University Hospital Oslo Norway; 2 Institute of Clinical Medicine Faculty of Medicine University of Oslo Oslo Norway; 3 Department of Cooperation, Patient Education and Equivalent Health Services Oslo University Hospital Oslo Norway; 4 Faculty of Health and Social Sciences University of South-Eastern Norway Drammen Norway; 5 Department of Psychiatry & Psychology College of Medicine & Science Mayo Clinic Rochester, MN United States; 6 Section of Specialized Endocrinology Department of Endocrinology, Morbid Obesity and Preventive Medicine, Division of Medicine Oslo University Hospital Oslo Norway; 7 Department of Medical Biochemistry, Institute of Clinical Medicine Faculty of Medicine University of Oslo Oslo Norway

**Keywords:** digital assessment, secure messages, patient portal, remote shared decision-making, chronic health conditions, assessment, portal, decision-making, chronic condition, chronic, communication, intervention, feasibility, pilot, acceptability, usage, demand, patient-reported outcome measures, PROM, outcome

## Abstract

**Background:**

Enhanced communication with health care providers (HCPs) can improve symptom management and health-related quality of life (HRQoL) for patients with chronic health conditions. Access to appropriate communication venues is needed to improve communication, however. As such, digital communication interventions mediated by patient portals carry the potential to support patient-provider communication and interaction and through this, also facilitate shared decision-making (SDM). The *InvolveMe* intervention was designed to provide patients with the opportunity to communicate symptoms and informational needs prior to consultation via digital assessment, including prioritizing what is most important to discuss with their HCPs, as well as to interact with HCPs through secure messages between outpatient visits.

**Objective:**

The aim of this study was to assess the feasibility of the *InvolveMe* intervention by investigating acceptability, demand (ie, system use), and limited efficacy.

**Methods:**

The study was designed as a single-arm, pre-post feasibility study combining quantitative and qualitative methods for data collection. Patients from an endocrine outpatient clinic were invited to use the *InvolveMe* intervention for 3 months, and HCPs administering *InvolveMe* were invited to participate in a focus group. Guided by descriptions of how to design feasibility studies by Bowen et al, feasibility was tested by exploring (1) acceptability, using data collected during recruitment from patient participants and nonparticipants (ie, declined to participate or did not meet study requirements), HCP experiences with recruitment, and the System Usability Scale (SUS); (2) demand via exploration of system use through extraction of system log data and HCP experiences with system use; and (3) limited efficacy testing, via exploration of potential effects from the Short-Form Health Survey (RAND 36), Hospital Anxiety and Depression Scale, and Health Literacy Questionnaire.

**Results:**

Patient participants (N=23) were a median 54 (range 26-78) years old and primarily male (14/23, 61%). Nonparticipants (N=16) were a median 73 (range 55-80) years old and primarily male (12/16, 75%). The average SUS score was 72.2, indicating good system usability. Assessments were completed by 8 participants from home prior to outpatient visits. The assessments entailed various bodily symptoms and needs for information. Participants sent 17 secure messages related to patient administrative matters, symptoms, and challenges. Focus group participants (N=4) were all female and registered nurses. Data were analyzed in 2 predefined themes: Acceptability and Demand. Acceptability included the subthemes intervention attractiveness and intervention suitability. Demand included the subthemes elements of SDM and intervention challenges and opportunities. All patient participants completed outcome measures at baseline, and 19 (19/23, 83%) completed outcome measures at 3 months. These preliminary efficacy findings were mixed and inconclusive.

**Conclusions:**

The study design provided findings from both patient and HCP perspectives and supported feasibility of the *InvolveMe* intervention. The investigation of acceptability and demand supported the potential for remote SDM mediated by patient portals using assessments and secure messages.

**Trial Registration:**

ClinicalTrials.gov NCT NCT04218721; https://www.clinicaltrials.gov/ct2/show/NCT04218721

## Introduction

Living with a chronic health condition is demanding, as chronic health conditions often cause a variety of symptoms (eg, anxiety, depression, fatigue, loneliness, and sleeping problems) that may negatively affect health-related quality of life (HRQoL) [1-4]. In order to manage the various symptoms they experience, patients need to be able to communicate and interact with health care providers (HCPs) [5,6]. However, experiences with poor communication and interaction between patients and HCPs are common, which may interfere with symptom management and help-seeking [7,8]. Shared decision-making (SDM) may, with its focus on the patient and HCP working together to understand and address the patient’s situation, carry the potential to improve patient-provider communication and interaction [9-11]. Traditionally, SDM has taken place in physical patient-provider encounters. However, information and communication technology (ie, eHealth) has provided new opportunities for SDM to be explored by improving access to care, enabling information exchange, supporting patient-provider communication, and building relationships [12].

Remote SDM may provide benefits by helping HCPs to understand which aspect of the patient’s problem requires action and, together with the patient, identify the action required to solve the problem [13]. Patient portals present one way to engage patients and providers in remote SDM [14]. A patient portal provides patients with secure online access to their own health information, such as HCP’s journal notes, medication lists, and opportunities for communication with their HCPs via secure messaging [15]. A review of patient portals [14] found that use of portals supported information sharing, improved preparation before visits, and supported patient-provider communication. Furthermore, portal use was found to encourage engagement in self-management of chronic disease [14,16] and empower patients in SDM [14]. However, the review rated the evidence related to portal use for improved communication, information sharing, and patient-provider relationships as low [14]. Secure messaging was identified as the most commonly reported portal feature, with patient-generated data by remote patient-reported symptoms (ie, assessments tools) less frequently reported [14].

Secure messages can also be an integral part of SDM, providing patients and HCPs with opportunities for contact, and benefits to patient-provider communication from using secure messages have been implied [17-19]. A review identified patients’ main triggers for sending secure messages as accessibility to HCPs, self-management, and unmet needs [20]. Furthermore, the review highlighted that consequences of patient-provider secure messaging included patient empowerment, health promotion, and acquisition of uncertain answers [20]. Another review, focusing on use of secure messages, reported improved or comparable patient health outcomes for patients with chronic conditions when using secure messages compared with in-person care, describing quality of care as equivalent or improved for chronic conditions [21]. Use of secure messages among cancer patients has also been associated with improved survival and reduced treatment-related admissions, as well as reduced emergency visits [22]. In addition, the use of secure messages has been associated with improved glycemic level among patients with diabetes [23].

Remote assessment in preparation for health care visits can support symptom management [24], which is an important and integral part of chronic health care. Collecting patient-reported symptom data remotely can provide an opportunity to address the individuality and variability in symptoms over time among patients. Remote collection of patient-reported symptoms can also make the clinical workflow more efficient by not requiring patients to complete assessments in the waiting room or report symptoms within the limited time for consultation with HCPs [24]. A recent review found that there were few published studies examining integrated systems (ie, more than one system act together as one) for remote patient-reported symptoms, primarily feasibility and pilot studies, and subsequently limited evidence exists related to care and outcomes from using such integrated systems [24]. However, results from standalone systems (ie, a system that functions independently of other systems) for remote patient-reported symptoms are promising. The use of such systems has been reported to reduce symptom burden [25-27] and decrease emergency visits and in-hospital admissions [28]. Also, a review found improved symptom control, HRQoL, patient satisfaction, and patient-provider communication when patient-reported symptoms were used in feedback to patients [29]. A review on the effectiveness of digital assessment tools to improve SDM [12] also found that communication, especially information sharing related to the patient’s HRQoL and social aspects, as well as provider management of the patient’s condition, improved through use. The review highlighted that digital assessment tools can be especially important for people with chronic health conditions [12].

Even though benefits of patient-provider communication and patient outcomes from the use of secure messages and remote patient-reported symptoms have been reported, research examining digital interventions combining secure messages and remote patient-reported symptoms through patients’ portals to facilitate SDM is scarce. There are also patient barriers to the use of patient portals, such as lack of user-friendliness, technical support, education, and access to the internet [16,30]. Patient age may also play a role [30], and tailoring digital patient-provider communication interventions through the involvement of stakeholders representing end users (eg, patients or HCPs) appears crucial [31,32]. Stakeholders can provide insight to help tailor interventions to suit the local context (eg, hospital setting) and thus make interventions more acceptable, user-friendly, and less complex [31,32]. There are several factors that may impact intervention implementation, both relating to population and individuals [33]. For example, adaptation and tailoring to context are acknowledged as important implementation strategies [34], and creating an understanding of the context in which the intervention will be used can hence help avoid development of interventions that may fail during evaluation [33].

Seeking to address some of the issues raised by existing research, the current research team designed and developed a digital patient-provider communication intervention, called *InvolveMe*, aiming to support patients living with chronic health conditions, such as patients with nonfunctioning pituitary adenomas (NFPA) [35]. This single-arm pilot study aimed to assess the feasibility of the *InvolveMe* intervention by exploring acceptability, demand (ie, system use), and limited efficacy using a combination of qualitative and quantitative methods.

## Methods

### The *InvolveMe* Intervention

The *InvolveMe* intervention was developed to support SDM in the follow-up of patients with chronic health conditions, by being tailored to suit the patient group [35]. The intervention was further tailored to suit the intended context (ie, endocrine outpatient clinic) [36], in this study, patients with NFPA. *InvolveMe* provides patients with the opportunity to remotely report symptoms, needs, and preferences for care by completing an assessment (ie, predefined symptom list) in the hospital’s patient portal. In addition, patients can use the secure messaging feature in the patient portal to interact with HCPs about symptoms and needs between hospital visits [35]. To allow for integration in patient portals, the *InvolveMe* assessment feature was developed as a Single Page Application (ie, web-technology) in line with the HL7 FHIR standard (ie, a specification for health care interoperability) [37]. The assessment part of *InvolveMe* is organized in 4 categories: (1) *bodily symptoms* (eg, pain, fatigue), (2) *psychosocial challenges* (eg, anxiety, loneliness), (3) *the need for work-related support* (eg, work-related understanding, whether the job exacerbates health), and (4) *the need for information* (eg, medication side effects, treatment change). The system allows for all symptoms and needs to be marked. In the first 3 categories, patients can rate how bothersome they find the symptom, while in *The need for information* category of the assessment, patients can request information from a predefined list. All symptoms and needs can be prioritized according to patients’ preferences for care, on a scale from 0 to 10. The completion of the assessment generates a summary that is sent to the patients’ HCPs (ie, as an attachment via the secure message feature).

**Figure 1:**
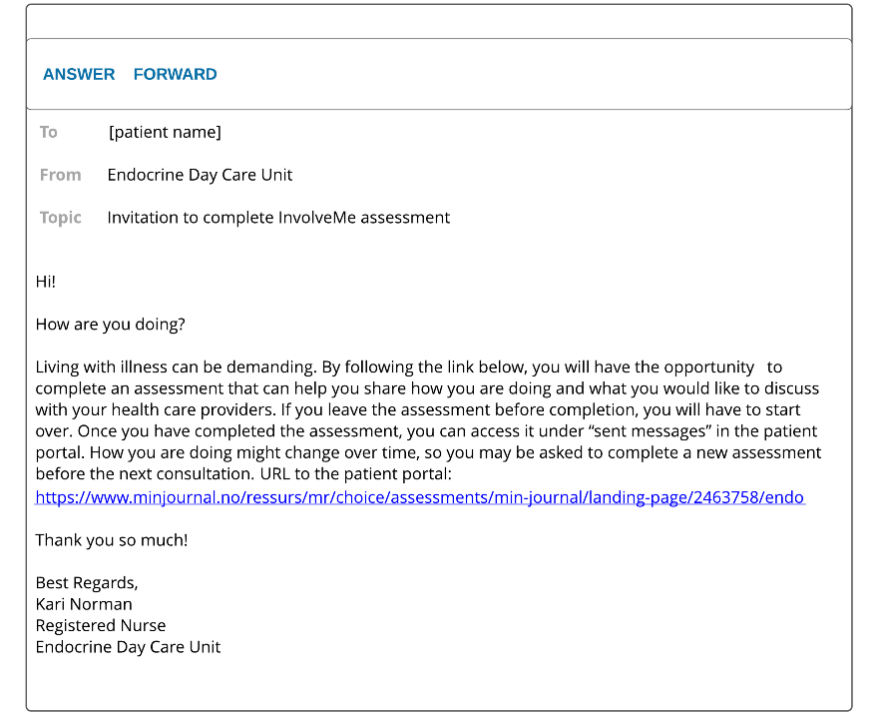
Invitation to complete the *InvolveMe* assessment from the patient interface.

**Figure 2:**
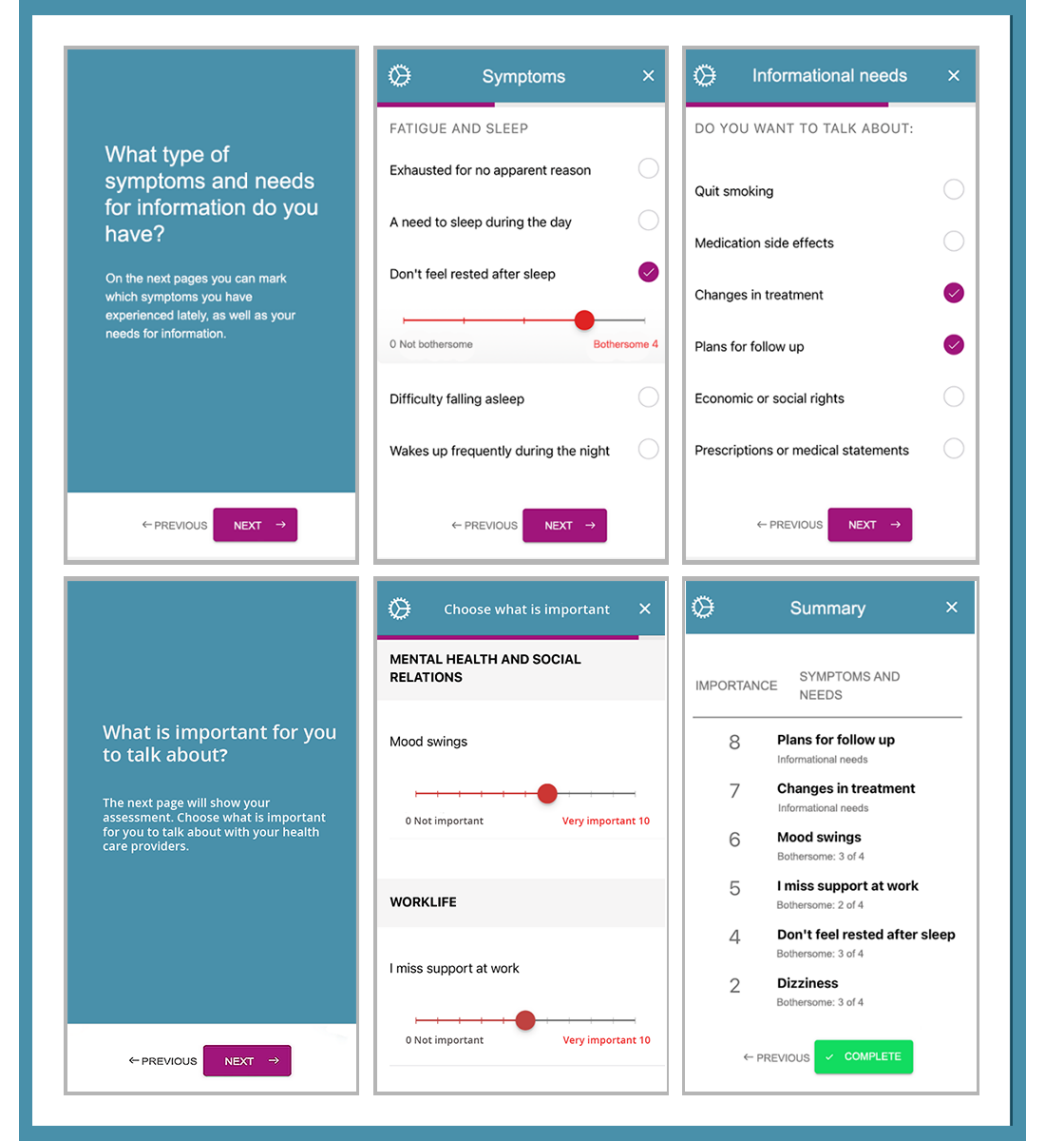
Screenshots of the *InvolveMe* assessment feature from the patient interface.

**Figure 3:**
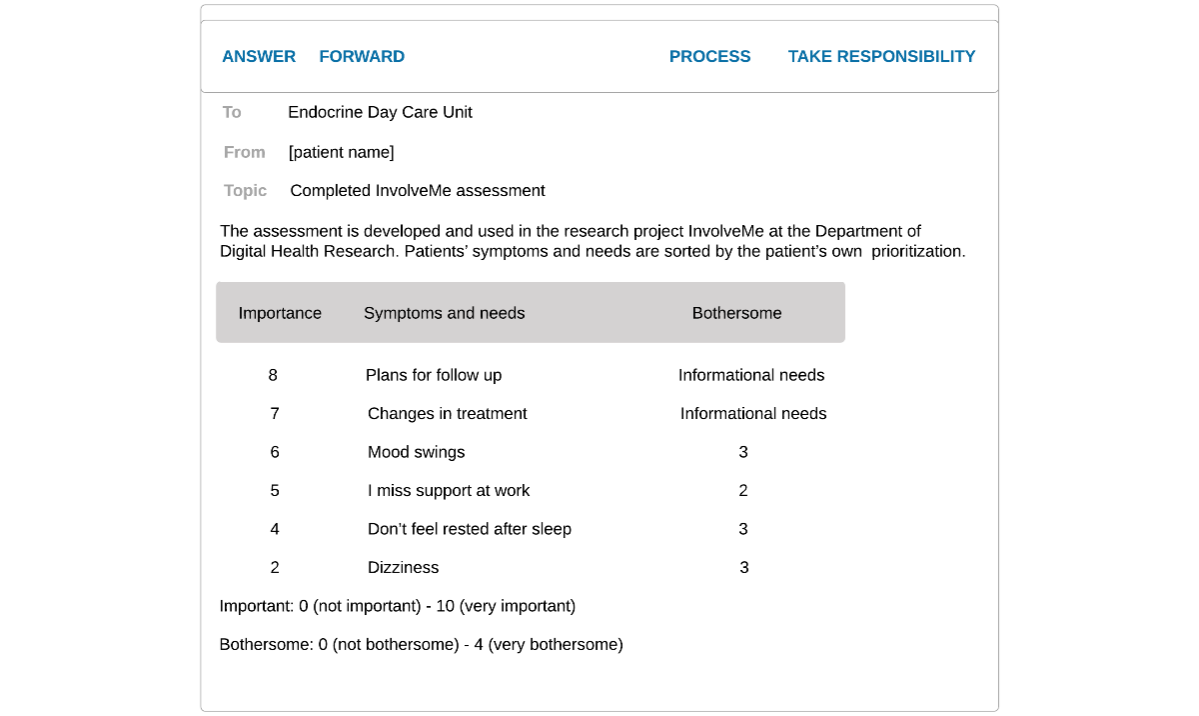
Completed assessment received from the health care professional (HCP) interface.

In this study, the assessment was used as preparation prior to upcoming in-person outpatient consultations, as well as for feedback during the consultations. Secure messages were sent from, and received in, a shared message inbox managed by a dedicated moderator (ie, registered nurses). Routines for the moderator were established in dialog with the registered nurses to suit daily clinical workflow. The moderator would send a secure message through the patient portal approximately one week prior to the planned visit with an invitation for patients to complete an assessment (See Figure 1). *InvolveMe* was accessed by patient participants through the hospital patient portal and could be used on smartphones, tablets, or PCs. The shared message inbox had an automated message response, providing patients with contact information in case of medical emergency, response time, and contact information for the endocrine outpatient clinic. See Figure 2 for selected screenshots of the *InvolveMe* assessment feature from the patient interface. HCPs accessed the shared message inbox through hospital computers. See Figure 3 for screenshots showing the HCP interface of a completed, received assessment. The *InvolveMe* intervention was provided as an addition to standard care.

### Study Design

In order to evaluate the effectiveness of complex interventions, initial testing and refinement of the intervention to ensure its feasibility are recommended [38]. Feasibility studies are important for producing findings that can be used to tailor interventions and examine recruitment settings [39]. This study was therefore designed as a pre-post feasibility study, with all patient participants receiving the *InvolveMe* intervention. In addition to the perspective of patients, the perspective of nonparticipants (ie, patients who declined to participate or did not meet study requirements) was included in this study to elaborate on potential barriers for use of eHealth interventions, which are of special interest for universal health care delivery, as provided in Norway. In addition, the perspective of HCPs was included to gain an understanding of intervention delivery and use in clinical practice. Feasibility conceptualization was guided by Bowen et al [39], exploring (1) acceptability (To what extent is *InvolveMe* judged as suitable, satisfying, or attractive?), (2) demand (Exploration of the actual use of the *InvolveMe* intervention and experiences with use from the HCP perspective), and (3) limited efficacy testing (Does the tool show promise of being successful with the intended population?) [39]. For the study purpose, this study combines quantitative and qualitative methods for data collection.

### Setting, Participants, and Recruitment

The participants in this study were patients with NFPA and HCPs recruited from an endocrine outpatient clinic at a university hospital in Norway. NFPA are benign pituitary tumors, with which patients frequently experience a long period of slow deterioration of their health status before undergoing surgery, and they usually experience a variety of symptoms in the aftermath [40-42], which negatively impacts HRQoL [41,42]. Patients experience individuality and variability in symptoms, including pain, fatigue, sleeping problems, anxiety, and depression, and they may also face challenges related to visual limitations, fear of recurrence, distressing thoughts, loneliness, and frustration [41,43]. Patients with NFPA need and receive long-term follow-up in outpatient care after surgery. The initiative to include this patient group came from the endocrine outpatient clinic participating in this study, which recognized a need to improve patient follow-up after surgery.

Eligibility criteria for inclusion of patients in the study were (1) a diagnosis of NFPA (anywhere in the disease trajectory); (2) receiving treatment and follow-up from the study endocrine outpatient clinic; (3) ≥18 years of age; (4) able to understand oral and written Norwegian; (5) access to a smartphone, tablet, or personal computer; (6) access to the internet with a secure access key (BankID).

Participating HCPs were registered nurses responsible for care and follow-up of NFPA patients at the endocrine outpatient clinic. Some of them had previously participated in studies related to the development and intervention tailoring of *InvolveMe* [35,36].

### Ethical Considerations

The study was approved by the Regional Committee for Medical and Health Research Ethics (2018/2201) and the Oslo University Hospital Institutional Review Board equivalent function (2017/9223). Informed consent was obtained from all participants.

### Study Procedure

Registered nurses and physicians at the endocrine outpatient clinic identified eligible patient participants based on study inclusion criteria, and the registered nurses asked if these patients were interested in receiving information about the study. Some of the patients were contacted and asked prior to upcoming consultations; others were asked during consultations. Those interested in receiving more information were contacted by the first author (BS) by phone and provided with information about the study purpose and procedures. Those interested in study participation signed a digital information and consent form. Patient-reported outcome measures were collected online through a secure server at Services for Sensitive Data (TSD; University of Oslo). After completing baseline outcome measurements, patient participants were contacted by the first author and informed how to register and log into the patient portal to access the *InvolveMe* features*.* After the first log on, HCPs sent a welcome message with information about the project to the patient participant. Patient and HCP participants could contact the first author by phone during the day on weekdays in case of questions. All contacts with participants were logged. The patient participants were informed to direct emergency issues or non-study-related questions to their primary care team or the nearest hospital or urgent care treatment unit.

HCPs at the endocrine outpatient clinic were provided with information about the study, and those willing to participate were included.

### Data Collection and Outcome Measures

#### Timeframe

Data collection from patient participants was carried out from April 2020 until October 2020, when an unexpected incident led to the closure of the hospital patient portal and subsequently closure of the study before the planned study period completion. Outcome measures were collected from patient participants prior to them receiving access to *InvolveMe* and after 3 months of access. Numbers of assessments and secure messages, as well as the content in the secure messages, were also collected. Data from HCP participants were collected through a digital focus group in December 2021.

#### Sociodemographics and Disease-Related Measures

Information about patient participants’ age, sex, level of education, work, income, and year of diagnosis and whether participants had received surgery were collected at baseline. HCP participants were all female registered nurses working in the endocrine outpatient clinic.

#### Acceptability—Patient Perspective

To explore to what extent the intervention was judged as satisfying or attractive, the first author’s experiences from introducing patient participants to the *InvolveMe* intervention, including registration and login procedures in the patient portal, were written down. In addition, patient participants completed the System Usability Scale (SUS), a 10-item survey that provides a comprehensive assessment of subjective usability [44], at the 3-month follow-up. The SUS is a widely used subjective rating tool with acceptable reliability and validity [45-47]. Data from patients who declined to participate in the study (ie, nonparticipants), including age, sex, and reason for not participating in the study, if given unsolicited, were collected.

#### Demand (System Use)—Patient Perspective

Details of actual system use of the *InvolveMe* intervention were extracted from the patient portal. These data included the number and content of secure messages sent by patient participants, number of assessment invitations sent from HCPs, and number of and content in the assessments completed by patient participants. In addition, reasons for noncompletion of assessments were collected by the first author by phone (ie, written down).

#### Acceptability and Demand—HCP Perspective

The HCP participants were invited to share their experiences in a focus group that was conducted digitally due to national in-person meeting restrictions during the COVID-19 pandemic. The focus group interview guide consisted of open-ended questions based on operationalization of acceptability and demand [39] after dicussions and consensus of the research team. To explore to what extent the intervention was judged as attractive, suitable, and satisfying (ie, acceptability), participants were asked questions about experiences with recruitment and system usability. To explore experiences with system use (ie, demand), participants were asked questions about the use of secure messages and assessments. The focus group was facilitated by the first (BS) and last (EB) authors, lasted 45 minutes, was recorded with a digital voice recorder, and was transcribed verbatim by the first author.

#### Limited Efficacy Testing

To explore the feasibility of outcome measures and whether *InvolveMe* could show promise of being successful with the intended population, as well as explore preliminary indications of the potential impact of using *InvolveMe*, participants completed the following outcome measures: anxiety and depression, HRQoL, and health literacy.

Anxiety and depression were measured with the Hospital Anxiety and Depression Scale (HADS), a 14-item measure of anxiety and depression [48]. Items were rated on a 4-point scale (0-3), with a total score ranging from 0 to 42. The HADS is divided into 2 subscales: anxiety (HADS-A; 7 items) and depression (HADS-D; 7 items).

HRQoL was measured with the noncommercial RAND 36 survey, a 36-item HRQoL measure of physical, emotional, cognitive, role and social functioning, physical health, and general and global health [49,50]. Scores can range between 0 and 100 for all subscales, with lower scores indicating higher disability (0=maximum disability, 100=no disability).

Health literacy was measured with the Health Literacy Questionnaire (HLQ) [51]. The 44-item questionnaire includes 9 independent scales, with each scale including 4 to 6 items. The first 5 scales (Part 1 of the HLQ) are scored using response options indicating the level of agreement to items (1=strongly disagree, 2=disagree, 3=agree, 4=strongly agree), while the 4 remaining scales (Part 2 of the HLQ) report on the capacities to undertake different tasks (1=cannot do or always difficult, 2=usually difficult, 3=sometimes difficult, 4=usually easy, 5=always easy) [51].

### Statistical Analysis

Statistical analyses were completed using SPSS version 25 (IBM Corp, Armonk, NY). Data on baseline characteristics and perceived usefulness are presented as medians and ranges for continuous variables and as proportions with percentages for categorical variables. Dependent paired *t* tests were used to analyze pre-post intervention changes. All tests were 2-sided, and *P* values <.05 were considered statistically significant.

### Qualitative Analyses

Data from secure messages sent by patient participants and data from the focus group with HCP participants were analyzed using thematic analysis inspired by Braun and Clarke [52]. The analysis process was led by the first author (BS) in close collaboration with the last author (EB). Data from 17 secure messages (ie, written text) were read by the first and last authors and coded inductively by the first author [52]. Quotes (ie, written text) to illustrate the content of the secure messages were then chosen by the first and last authors and discussed within the research team.

The first and last authors read the transcript from the HCP focus group to become familiarized with the data [52]. Then, the 2 authors used the interview guide to code the transcript deductively into 2 predefined codes: (1) acceptability and (2) demand. Next, subthemes within each main theme were identified. Themes and subthemes were then re-examined, and quotes to illustrate each subtheme were finally chosen and discussed within the research team [52].

## Results

### Recruitment, Participant Flow, Sample Description

**Figure 4:**
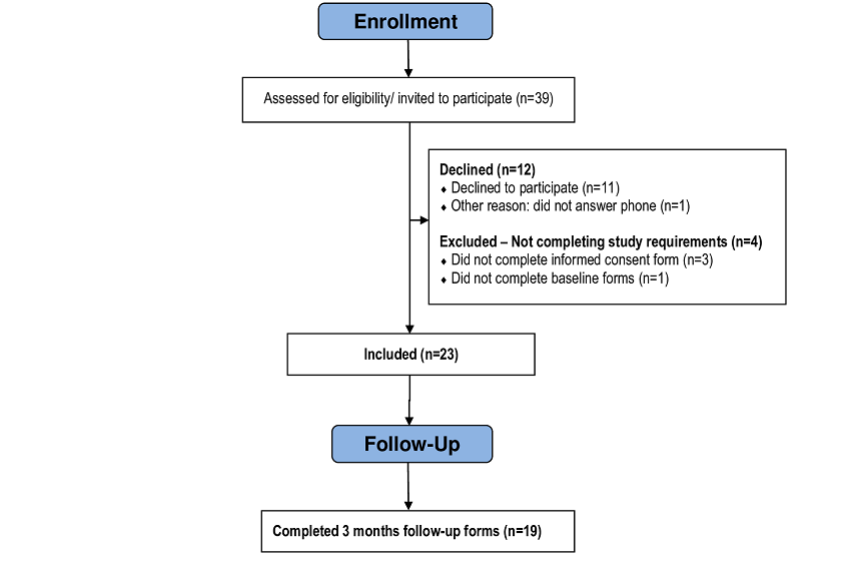
Recruitment flowchart.

Of 39 patients with NFPA who were assessed for eligibility, 23 (59%) agreed to participate (ie, patient participants), and 16 (41%) declined or did not meet study requirements (ie, nonparticipants). The 23 participants who were included in the study completed baseline measures and received the *InvolveMe* intervention. Of these, 19 participants completed the 3-month follow-up outcome measures. Due to technical issues with the hospital patient portal, the study closed after 6 months, which meant that 4 of the final included participants had limited time (ie, 1 to 4 weeks) to use *InvolveMe*. These 4 were hence not invited to complete the 3-month follow-up outcome measures. One of these participants completed and returned a secure assessment, but none of them used the secure message option in *InvolveMe*. Figure 4 provides details of the study recruitment and participant flow.

Patient participants (N=23) were a median 54 (range 26-78) years old at inclusion. Of these, 17 (74%) had completed surgery. Participants were mostly male (14/23, 61%), and almost one-half (10/23, 44%) noted elementary school as their highest level of education (see Table 1 for details). The nonparticipants (N=16) were a median 73 (range 55-80) years old and mostly male (12/16, 75%). HCP participants (N=4) were all female and registered nurses working at the endocrine outpatient clinic.

**Table 1:** Patient participants' baseline demographics and illness characteristics (N=23).

Characteristics			Results
Age (years), median (range)			54 (26-78)
**Sex, n (%)**			
	Female		9 (39)
	Male		14 (61)
**Marital status, n (%)**			
	Married/cohabitating		15 (65)
	Single/divorced		8 (35)
**Education, n (%)**			
	Elementary/high school		10 (44)
	University/college ≤4 years		7 (30)
	University/college >4 years		6 (26)
**Employment status, n (%)**			
	Full-time/part-time work		7 (30)
	Sick leave/disability benefits		10 (44)
	Retired/other		6 (26)
**Income (NOK^a^), n (%)**			
		200,000-399,999	6 (26)
		400,000-599,999	7 (30)
		600,000-799,999	0 (0)
		800,000-1,000,000	2 (9)
		>1,000,000	8 (35)
Surgery, n (%)			17 (74)
Months since surgery^b^, median (range)			11 (1-39)

^a^NOK: Norwegian krones; a currency exchange rate of NOK 1=US $0.90 is applicable.

^b^n=17.

### Acceptability—Patient Perspective

Among the 23 patients receiving the *InvolveMe* intervention, 7 (30%) needed additional technical support and assistance beyond the 2 planned contacts during study inclusion to be able to complete study requirements (ie, to complete the digital consent form, complete the digital baseline forms, or register in the patient portal). There were no questions from participants related to completing outcome measures after the initial guidance on how to complete the digital forms. At the 3-month follow-up, the 19 participants also completed the SUS. Mean system usability (ie, SUS) score was 72.2 (SD 14.6), which equals good system usability [44]. Of the 16 nonparticipants, 4 (25%) were positive toward participating but did not complete study requirements such as informed consent or baseline outcome measures. Reasons for declining were mainly described as not having a smartphone (7/16, 44%), feeling overwhelmed (2/16, 13%), or that they thought the intervention would be difficult to use (2/16, 13%). Some stated that it could be challenging to participate in a digital intervention, and some were not familiar with certain terms such as “smartphone.”

### Demand (System Use)—Patient Perspective

During the 3-month study period, 43% (10/23) used the *InvolveMe* intervention (ie, used the secure message, completed the assessment, or both) before study closure.

#### Secure Messages

Of the included patient participants, 4 (4/23, 17%) sent a total of 17 secure messages (ie, assessments not included) during the study. HCPs responded to all, mainly by messages, some by phone or in-person in the upcoming consultation. The content of the messages from participants were sorted into 2 codes: (1) Patient Administrative Matters and (2) Symptoms and Challenges.

For the “Patient Administrative Matters” code, the 9 secure messages mainly concerned a change in the scheduled time of a hospital appointment, prescriptions for medications, or other practical matters. One participant wrote:

I have called the pharmacy for a while, but they have not received my medicine. Think the medicine is called something like [medication name]. I was advised by the doctor to take it during my appointment in May. Have called you too, but no answer.

Participant 11

For the “Symptoms and Challenges” code, the 8 secure messages concerned various symptoms and challenges experienced by the participants. The messages centered on a need for guidance (ie, including information and advice) regarding how to manage various symptoms and how to live with the chronic health condition. One participant wrote:

What should I feel or look for when I work with challenging things over time, or to see the degree to which I can work-out. Previously I have been told to double the dose of [medication] when needed, but when is that? What are the risks associated with the procedures that have been performed?

Participant 5

Another participant wrote:

I am still on sick leave, as I feel VERY and UNUSUALLY tired. I get easily tired after doing something. Have also had strict restrictions about making sure I take it easy, not bending forward, sleeping in at least a 30 degrees upwards position, not just showering hot, not eating hot/spicey food etc. (...) Have today raised hemoglobin to [X], as I have lost a lot of blood which may have affected the situation? (...) I’m not quite sure what to do to feel better?

Participant 16

#### Assessments

HCPs sent invitations to complete intervention assessments to all patients with upcoming consultations. Of the 13 invitations sent, 8 (62%) participants completed the assessments prior to the scheduled consultation. In a phone conversation, 1 patient said about the assessment:

So incredibly beneficial to be enabled to meet prepared.

Statement, Participant 17

Patient participants marked their symptoms and needs in all 4 assessment categories. The categories *Bodily symptoms* and the *Need for information* were marked in all assessments. The most prevalent need for information was about the disease trajectory, marked by 7 (7/8, 88%) participants. The number of marked symptoms and needs varied from 3 to 17 (median 6.5). See Table 2 for an overview of content in the completed assessments.

**Table 2:** Overview of the content in completed assessments (n=8).

Assessment main categories	Completed individual assessments							
	1	2	3	4	5	6	7	8
								
Bodily symptoms	Y^a^	Y	Y	Y	Y	Y	Y	Y
Psychosocial challenges	N^b^	Y	Y	Y	Y	N	Y	Y
The need for work related support	N	N	Y	N	N	N	Y	N
The need for information	Y	Y	Y	Y	Y	Y	Y	Y

^a^Y: yes.

^b^N: no.

Reasons for not completing the assessment varied: The upcoming consultation was rescheduled; the assessment was not received by the user due to technological difficulties; and one participant “felt fine” and felt no need to complete an assessment. Two participants did not receive an assessment notification in the patient portal and were therefore not aware of the assessment invitation. Patients had to register their contact information in the hospital patient portal in order to receive notifications there, and a failure to do so could potentially explain why these participants did not receive a notification.

### Acceptability and Demand—HCP Perspective

Findings from the focus group with HCP participants were analyzed into the 2 predefined themes of Acceptability and Demand.

#### Acceptability

Participating HCPs provided a variety of feedback on the intervention, constituting 2 subthemes: (1) Intervention Attractiveness and (2) Intervention Suitability.

In the Intervention Attractiveness subtheme, HCP participants described their experience with the intervention in favorable words and phrases. They described the availability that the intervention provided for the patients as favorable, being able to contact HCPs when they needed to. They also stated that the intervention provided a unique option, especially for patients with complex health issues or heavy symptom burden. They stated that they would have liked to use the intervention for a longer period than the actual study period and said they would like to be a part of and use the intervention in a potential future clinical trial. Regarding recruitment of patient participants, the HCPs described most patients as interested and easy to recruit for this study. As one HCP stated: “...it was not difficult to recruit patients at all, they were, many were positive...”

In the Intervention Suitability subtheme, participating HCPs described the need for a secure and safe place for patients and HCPs to be able to communicate digitally and stated that the intervention was suited for this purpose. They highlighted that their, as well as the patient participants’, previous involvement in the process of intervention development and tailoring was an important factor to make the intervention suited to purpose. However, based on experiences of recruiting participants for the pilot study, the HCPs were not entirely convinced that the intervention was suitable for older patients. One HCP stated that older patients sometimes lacked the necessary equipment (eg, a smartphone) to participate, and reflecting on this, other HCPs contemplated whether a pre-educational group could be useful for eligible patient participants that needed guidance on how to use the intervention. The participating HCPs described themselves and the participating patients as satisfied with the *InvolveMe* intervention and described how patients had provided positive feedback to them about the intervention. One HCP stated that “...the participating patients gave the impression of being very satisfied, they thought it was exciting, and nice, that they could send questions.”

#### Demand

The participating HCPs described the actual use of the *InvolveMe* intervention, and findings constituted 2 subthemes: (1) Elements of SDM and (2) Intervention Challenges and Opportunities.

In the Elements of SDM subtheme, HCPs described how the assessment helped patient participants sort their thoughts before the hospital visits and stated that it acted as a way of providing information about common symptoms and needs. One HCP described the assessments completed prior to consultation as making it easier to address sensitive topics in in-person conversations with patients. This was supported by the other participating HCPs. Some of them described how patients’ identification and prioritization of topics important to them contributed to a focus in the consultation conversation, centering around what was most important to discuss for the patients. The HCPs described how the assessments had contributed to change their perspective on what was important to discuss with patients and said that, through this information exchange focusing on patients’ current situations, a more individualized follow-up was facilitated based on the patient’s needs. As described by one of the HCPs:

...the most important thing is that it is user centered, that patients dare to raise issues that are important to them, so that we can focus on what is important, for them to benefit the most from the health care service, this is very important and very rewarding.

Also, one HCP described how the intervention provided support and contact for patients who felt unprepared for the aftermath of surgery. This was also supported by the other participating HCPs.

In the Intervention Challenges and Opportunities subtheme, participating HCPs described the intervention as time-consuming initially, as they had to learn a new system (ie, hospital patient portal) and develop new routines to be adapted into the daily clinical workflow. However, they stated that, after having learned to use the system, it was no longer time-consuming but rather something that could be executed in between other daily tasks. As stated by one HCP:

...we had to learn a new system, which we spent some time on, but I think it was quite easy to learn the system, and it quickly became the routine.

One participating HCP also stated that they spent some time between themselves discussing potential responses to patients before replying to the secure messages from patients. They reported considering this as something positive, providing quality-assured responses to patients and also contributed to the development of care though contributing to professional discussions. One participant stated that the assessment could potentially even provide support for new HCPs with little prior knowledge about the patient group. They also highlighted, based on feedback from patients, that access to the intervention was valued, even by the patients not using the intervention (ie, participating nonusers), stating that interventions were often used the most by those with complex health issues or heavy symptom burden.

### Limited Efficacy Testing: Pre-Post Intervention Results

Pre-post intervention findings at the 3-month follow-up revealed statistically significant increases in symptoms of anxiety (mean difference [MD] 3.9, 95% CI 2.3-5.5) and depression (MD 2.7, 95% CI 0.9-3.8) for the participating patients. Time since surgery had no impact on these results. HRQoL findings indicated a statistically significant improvement for the “Role Physical” subscale (MD 25.0, 95% CI 3.0-47.0) but not for the 7 other subscales. There was a high degree of heterogeneity in the data, with large variance and subsequently broad CIs for the HRQoL subscales (eg, the “Role Emotional” subscale improved by 17.5, but due to the large variance, the findings were not statistically significant). Scores related to health literacy remained stable, with no statistically significant changes from baseline to follow-up. See Multimedia Appendix 1 for details.

## Discussion

### Principal Findings

Findings from this feasibility pilot study gave insights related to the acceptability and demand of the *InvolveMe* intervention. Exploration of intervention acceptability identified good system usability, and the findings also provided insights regarding patients’ reasons for not participating, as well as demographic factors impacting intervention participation (eg, older age among nonparticipants). Furthermore, some participants appeared to struggle with understanding terms used to describe study participation. The examination of demand (ie, system use) suggests that completed assessments and use of secure messages may respectively act as preparation for upcoming visits and provide patients with the opportunity to request guidance on symptom management. The limited efficacy testing showed mixed findings in terms of HRQoL, anxiety, and depression but indicated a study population with high health literacy.

This study provided insight into opportunities for remote SDM through use of secure messages and digital assessments mediated by a patient portal. During the study period, only 17% of participants used the secure message feature. However, simply having the access and opportunity to communicate with HCPs may be of benefit to patients, even without using this option [53]. This was as expected and also suggested in focus group with HCPs. About half of the secure messages sent in the study contained questions related to symptom and treatment complication guidance, which may indicate that the secure messages were used by those who experienced a heavy symptom burden at the time. This was also pointed out by the HCPs during the focus group.

Existing research has found patients to be interested in using secure messages with their HCPs and that patients prefer the convenient and asynchronous aspects provided by secure messaging through portals [14]. The modest use of secure messages in the current study could have been due to the brief study timeframe, and providing patient access over a longer period of time could have provided increased knowledge and experience related to the use of secure messages. Some patients may also lack interest in communicating through portals as they are satisfied with the existing in-person communication [14]. Health literacy is another factor that may play a role in patient portal use, as research has pointed to patients with lower literacy skills as being less likely to use patient portals [14]. However, the participants in this study did not have low literacy skills, quite the contrary.

The assessments collected prior to consultation in this study revealed a wide range of symptoms and needs experienced by the patient participants, which may help identify important topics and priorities for patient-provider discussions and SDM, as also pointed out in the focus group with HCPs. Such assessments may address the individuality and variability in symptoms, aiding patients with communicating their symptoms, needs, and preferences for care to their HCPs [54,55]. In all completed assessments, the study participants used the opportunity provided to request information. This could help improve the provision of tailored information to suit the patients’ situations and thus support patients in making choices about their lifestyle, when ready to do so. It has been suggested that, when patient preferences are asserted, HCPs may manage patient concerns and health conditions more effectively [12]. In line with SDM, the *InvolveMe* assessment feature, providing insight into patients’ current situations, may facilitate collaboration between the patient and provider to mutually understand and address the patient’s situation [9-11].

In this study, 41% of the eligible patients declined to participate or did not complete study requirements (ie, nonparticipants), which is a low percentage of people declining compared with similar studies examining eHealth interventions (ie, 60%-68%) [56,57]. However, evidence on how patients accept eHealth interventions is limited [58], and increasing knowledge related to reasons for nonparticipation (eg, user friendliness, complexity) is necessary in order to improve intervention acceptance.

Nonparticipants in this study were older (median 73 years) than the participants (median 54 years), corresponding with findings from existing research [56,59]. Increased age has been described as contributing to lower levels of digital skills [60,61], a known barrier for adopting new technology [30,62]. Along these lines, a review pointed to substantial health equity disparities in patient portal use, where older persons, persons with low socioeconomic status, persons with low health literacy, and persons with chronic health conditions appear to use portals less often [62].

In this study, some of the eligible patient participants struggled to understand some of the terms used to describe the study participation in detail. Use of technology may inadvertently create health equity concerns by not paying sufficient attention to the social determinants of health during the implementation process [62]. Instead of focusing on barriers for portal use, which may place responsibility on patients already experiencing health disparities, one should focus on developing interventions that are easy to use in order to reduce disparities [62,63]. Findings from this study, as well as existing research on strategies to minimize potential disparities in use [14,63,64], point to the need to develop strategies to increase the number of participants in future studies.

System usability was rated as good but not excellent in this study, which indicates room for intervention improvement. However, using the SUS [44] to measure usability may not have been ultimate in this study, as participants most likely rated the overall system usability, including all the features of the hospital patient portal, not the specific features of the *InvolveMe* intervention alone. When aiming to measure usability and evaluate features integrated into an existing system, the SUS may not be specific enough, and other or additional usability measures should be considered.

The psychosocial outcome measures in this study were primarily included to test feasibility of the measures (eg, are they easy to answer digitally, do they capture changes). Even though some participants initially struggled with completing the outcome measures, all 19 participants receiving the 3-month follow-up measures completed these. There were no questions from the participants related to outcome measures after the initial guidance on how to complete these, indicating satisfactory study routines for this aspect. The noted statistically significant increases in anxiety and depression during the 3-month study period were unexpected. These findings could however be related to the ongoing pandemic during this study, and a recent study revealed that the general population was almost 3 times more likely to suffer from symptoms of anxiety and depression due to the pandemic [65]. The current study period coincided with a national decrease in COVID-19 cases around baseline (ie, May 2020 to June 2020) and a national increase in cases around the 3-month follow-up (ie, August 2020 to September 2020), which might explain the pre-post increase in symptoms of anxiety and depression. However, given the feasibility nature as well as the limited number of participants in the study, efficacy conclusions cannot be made.

Compared with indicators of health literacy (ie, *Active engagement with HCPs* and *Read and understand health information*) from a population-based survey (ie, including people with chronic health conditions) [8] as well as general population participants [66], health literacy scores from this study indicate a study population with high health literacy. Reasons for these findings are not evident, although participants in this study were younger compared with nonparticipants, and higher age has been associated with lower health literacy [60,67].

Even though the closure of the hospital patient portal during this study caused premature study closure, the software development of the *InvolveMe* assessment feature is in accordance with a standard enabling the completed assessment to be sent as an attachment via the secure message feature in the national patient portal [68] as well. Use of standards for provision of eHealth systems has been recognized as a key factor for successful implementation [31,32], and the importance of developing new software features and systems according to established standards are clearly emphasized through this feasibility pilot study.

The COVID-19 pandemic brought an urgent need for remote care through secure, technical systems and as such, boosted the use of the national patient portal [68] in various ways. For example, as of March 2020, all COVID-19 test results were accessible to Norwegian citizens through the national patient portal, and a number of HPCs, including general practitioners, began using the national portal for most nonurgent care and follow-up. This increase in use of eHealth systems as a consequence of the pandemic has also been identified through a recent review, highlighting how the transformation of care from in-person to virtual or remote accelerated during this time [69]. The *InvolveMe* intervention, incorporated into the national patient portal, may provide features currently not used in the national portal, further promoting patient-provider communication and interaction and serving the need for remote care systems.

### Study Limitation and Strengths

This study has several limitations. First, the study was designed to assess the feasibility of a digital patient-provider communication intervention to support patients with NFPA. All patient participants received access to the intervention, without randomization, and statements regarding the effectiveness of the intervention cannot be made. Efficacy testing was however not a major part of this feasibility pilot study. Second, the participants were recruited through a collaborating partner (ie, endocrine outpatient clinic), and it may therefore be assumed that the participating sample were highly motivated and the study cannot conclude whether patients with NFPA in general would be interested in, or benefit from, such an intervention. Indications on feasibility are however promising. Third, the urgent closure of the hospital patient portal led to an unpredictably shortened study period, which might have affected study outcome. Fourth, the focus group with HCPs was conducted 1 year after the pilot study was finished, and all HCP participants were registered nurses. This might have affected recall of experiences, and other additional professionals could have elaborated even more on questions asked in focus group.

This study also has several strengths. First, both patient and HCP perspectives are included in the study, underlining the importance of stakeholder involvement when aiming for real world implementation [30-32]. Second, all eligible patients were invited for participation. This provided insight into who would be interested in the opportunity to assess symptoms and information needs prior to consultations and use secure messages to communicate digitally with HCPs between consultations, as well as reasons for nonparticipation. Such information could be used to tailor educational material and study routines for participant follow-up during the study. Third, the data collection related to acceptability and demand provided essential information for tailoring of the *InvolveMe* intervention as well as study routines in preparation for a future clinical trial.

### Future Directions

Through exploration of acceptability, demand (ie, system use), and limited efficacy testing, this study established feasibility of the digital patient-provider communication intervention *InvolveMe*. Findings provided ideas and suggestions for further tailoring in order to prepare for a future clinical trial, such as the development of study-specific questions (ie, in addition to SUS) [44]; use of simple, plain language in the recruitment processes and patient education material; as well as having a dedicated support person involved in the study. In the study, some participants struggled with completing study requirements. Future research should aim to incorporate ways to help adults not familiar with technology to become familiar with and adopt digital interventions.

The increasing number of persons living with chronic health conditions entails, in addition to individual personal challenges, increases costs and demands for resources, representing a major challenge for health care services. Therefore, future research should continue to explore how assessments and secure messages mediated through patient portals can promote and support remote SDM in a variety of chronic health conditions.

Finally, in order to examine actual effects of digital patient-provider communication interventions such as *InvolveMe*, larger-scale clinical trials are needed.

### Conclusions

This feasibility pilot study explored how a digital patient-provider communication intervention, *InvolveMe*, could be of use for patients living with chronic health conditions, such as patients with NFPA. Feasibility was established, and the importance of developing software according to given standards was highlighted. Given the findings showing that patient participants used the secure assessment and messages to communicate about bodily symptoms, needs for information, and challenges they experienced, the use of patient-provider interventions such as *InvolveMe* has the potential to facilitate SDM by enhancing accessibility and information exchange and to strengthen the patient-provider relationship for patients living with chronic health conditions.

## Appendix

Multimedia Appendix 1 Pre-post intervention changes.

## Abbreviations

HADS: Hospital Anxiety and Depression Scale
HCP: health care provider
HLQ: Health Literacy Questionnaire
HRQoL: health-related quality of life
MD: mean difference
NFPA: nonfunctioning pituitary adenomas
SDM: shared decision-making
SUS: System Usability Scale
