# Supplementary material for: Feasibility of a Digital Patient–Provider Communication Intervention to Support Shared Decision-Making in Chronic Health Care, InvolveMe: Pilot Study
Source: JMIR Form Res. 2022 Apr 7;6(4):e34738. doi: 10.2196/34738 (PMC9030980; doi:10.2196/34738)
Supplement: Multimedia Appendix 1 [file formative_v6i4e34738_app1.docx]

**Pre-post intervention changes in anxiety, depression, HRQoL and health literacy (n=19)**

| Psychosocial outcomes | | Baseline | | 3-month follow-up | | Mean difference | (95% CI of diff) | *P* value |
| --- | --- | --- | --- | --- | --- | --- | --- | --- |
|  | | Mean | (SD) | Mean | (SD) |  |  |  |
| **Anxiety** (HADS-A^a^) | | 4.4 | (3.0) | 8.3 | (2.8) | 3.9 | (2.3 to 5.5) | <.01 |
| **Depression** (HADS-D^b^) | | 3.1 | (2.5) | 5.5 | (2.2) | 2.7 | (0.9 to 3.8) | <.01 |
| **HRQoL**^c^ **(RAND-36**^d^**)** | |  |  |  |  |  |  |  |
|  | Physical function | 82.9 | (14.4) | 87.3 | (12.6) | 4.5 | (-2.7 to 11.6) | .20 |
|  | Role physical | 50.0 | (36.3) | 75.0 | (31.1) | 25.0 | (3.0 to 47.0) | .03 |
|  | Bodily pain | 69.7 | (22.9) | 77.0 | (20.6) | 7.2 | (-4.3 to 18.7) | .20 |
|  | General health | 59.7 | (18.4) | 65.5 | (15.2) | 5.7 | (-1.8 to 13.4) | .13 |
|  | Vitality | 55.8 | (23.4) | 60.5 | (22.0) | 4.7 | (-4.7 to 14.2) | .31 |
|  | Social function | 77.0 | (28.6) | 79.6 | (23.3) | 2.6 | (-12.9 to 18.1) | .73 |
|  | Role emotional | 68.4 | (39.2) | 86.0 | (33.9) | 17.5 | (-7.8 to 42.9) | .16 |
|  | Mental health | 80.0 | (14.7) | 78.3 | (19.5) | -1.7 | (-11.2 to 7.8) | .71 |
|  | Change | 47.4 | (31.1) | 60.5 | (31.5) | 13.2 | (-0.4 to 26.7) | .06 |
| **Health Literacy (HLQ**^e^**)** | |  |  |  |  |  |  |  |
|  | Healthcare provider support | 3.0 | (0.4) | 3.0 | (0.5) | 0.00 | (-0.2 to 0.2) | .89 |
|  | Having sufficient information | 2.7 | (0.4) | 2.8 | (0.4) | 0.1 | (-0.0 to 0.2) | .10 |
|  | Actively managing health | 2.8 | (0.4) | 2.9 | (0.6) | 0.1 | (-0.2 to 0.3) | .57 |
|  | Social support | 2.8 | (0.5) | 2.9 | (0.6) | 0.1 | (-0.1 to 0.3) | .39 |
|  | Critical appraisal | 2.5 | (0.6) | 2.5 | (0.5) | 0.0 | (-0.2 to 0.3) | .85 |
|  | Active engagement with HCPs | 3.7 | (0.7) | 3.7 | (0.6) | 0.0 | (-0.1 to 0.2) | .61 |
|  | Navigating the healthcare system | 3.5 | (0.8) | 3.6 | (0.6) | 0.0 | (-0.2 to 0.3) | .72 |
|  | Ability to find good health information | 3.6 | (0.6) | 3.7 | (0.5) | 0.1 | (-0.1 to 0.3) | .32 |
|  | Reading and understanding health information | 3.9 | (0.5) | 4.0 | (0.4) | 0.1 | (-0.1 to 0.3) | .31 |

^a^HADS-A: Hospital Anxiety and Depression Scale-Anxiety subscale (score range 0-21; a higher score indicates a higher degree of anxiety).

^b^HADS-D: Hospital Anxiety and Depression Scale-Depression subscale (score range 0-21; a higher score indicates a higher degree of depression).

^c^HRQoL: health-related quality of life.

^d^RAND-36: RAND 36-Item scale (score range 0-100; a higher score indicates higher emotional well-being).

^e^HLQ: Health Literacy Questionnaire (First 5 sub-scales: score range 1-4. Last 4 subscales; score range 1-5. A higher score indicates better health literacy).
